# Impact of sex on diabetic nephropathy and the renal transcriptome in UNx db/db C57BLKS mice

**DOI:** 10.14814/phy2.14333

**Published:** 2019-12-25

**Authors:** Frederikke E. Sembach, Lisbeth N. Fink, Thea Johansen, Brandon B. Boland, Thomas Secher, Sebastian T. Thrane, Jens C. Nielsen, Keld Fosgerau, Niels Vrang, Jacob Jelsing, Tanja X. Pedersen, Mette V. Østergaard

**Affiliations:** ^1^ Gubra ApS Hørsholm Denmark; ^2^ Department of Clinical Medicine University of Copenhagen Copenhagen Denmark

**Keywords:** albuminuria, animal model, diabetic nephropathy, mesangial expansion, uninephrectomy

## Abstract

Diabetic nephropathy (DN) is associated with albuminuria and loss of kidney function and is the leading cause of end‐stage renal disease. Despite evidence of sex‐associated differences in the progression of DN in human patients, male mice are predominantly being used in preclinical DN research and drug development. Here, we compared renal changes in male and female uninephrectomized (UNx) db/db C57BLKS mice using immunohistochemistry and RNA sequencing. Male and female UNx db/db mice showed similar progression of type 2 diabetes, as assessed by obesity, hyperglycemia, and HbA1c. Progression of DN was also similar between sexes as assessed by kidney and glomerular hypertrophy as well as urine albumin‐to‐creatinine ratio being increased in UNx db/db compared with control mice. In contrast, kidney collagen III and glomerular collagen IV were increased only in female UNx db/db as compared with respective control mice but showed a similar tendency in male UNx db/db mice. Comparison of renal cortex transcriptomes by RNA sequencing revealed 66 genes differentially expressed (*p* < .01) in male versus female UNx db/db mice, of which 9 genes were located on the sex chromosomes. In conclusion, male and female UNx db/db mice developed similar hallmarks of DN pathology, suggesting no or weak sex differences in the functional and structural changes during DN progression.

## Graphical abstract

Despite evidence of sex‐associated differences in the progression of DN in human patients, male mice are predominantly being used in preclinical DN research and drug development. Here, we compared renal changes in male and female uninephrectomized (UNx) db/db C57BLKS mice. We find that male and female UNx db/db mice developed similar hallmarks of DN pathology.

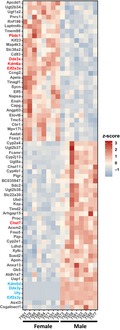

## 1 INTRODUCTION

The progressive rise in diabetes mellitus worldwide is paralleled by severe complications including retinopathy, neuropathy, and nephropathy (Valencia & Florez, 2017; Zhou et al., 2016). Approximately 40% of diabetic patients develop diabetic nephropathy (DN) (Reutens, 2013; Saran et al., 2018), which is characterized by albuminuria and loss of kidney function (Alicic, Rooney, & Tuttle, 2017; Reutens, 2013). DN is the leading cause of chronic kidney disease (CKD) and end‐stage renal disease (ESRD) accounting for around half of all patients entering dialysis, and is associated with lower quality of life, increased cardiovascular risk, and reduced survival (Saran et al., 2018).

Except for the recent emergence of SGLT2 inhibitors and GLP‐1 receptor agonists (Hocher & Tsuprykov, 2017) for nephroprotection in patients with diabetes, the DN therapeutic landscape has remained largely unchanged in the past 25 years (Johnson & Spurney, 2015; Wanner et al., 2016). This gap is largely due to the absence of reliable markers of disease risk and progression, as well as an incomplete understanding of the underlying mechanisms of DN in preclinical models and man. Thus, the development of animal models that more closely resemble the pathophysiology of human DN is urgently needed to support the advancement of novel and efficient therapies.

Several potential animal models of DN have been explored (Kitada, Ogura, & Koya, 2016). Notably, the db/db mouse which carries a homozygous inactivating leptin receptor mutation resulting in defective leptin signalling (Chen et al., 1996; Lee et al., 1996) and consequently hyperphagia, obesity, insulin resistance, and hyperglycaemia (Lee & Bressler, 1981) have been extensively utilized (Tesch & Lim, 2010). These mice develop many of the pathological features of early human DN, including albuminuria and mesangial matrix expansion (Cohen, Lautenslager, & Shearman, 2001), but only mild glomerulosclerosis. Accordingly, to accelerate the progression of DN, and hence to more closely mimic human pathophysiology, unilateral nephrectomy can be applied leading to additional hemodynamic stress on the remaining kidney (Bower, Brown, Steffes, Vernier, & Mauer, 1980).

Despite evidence of sex‐associated differences in the progression of DN in human patients (Carrero, Hecking, Chesnaye, & Jager, 2018; Chang et al., 2016; Piccoli, Alrukhaimi, Liu, Zakharova, & Levin, 2018), male mice are predominantly being used in preclinical DN research and drug development including the majority of previous studies in the UNx db/db mouse model of DN (Ninichuk, Khandoga, et al., 2007; Ninichuk, Kulkarni, Clauss, & Anders, 2007; Senador, Kanakamedala, Irigoyen, Morris, & Elased, 2009; Yiu et al., 2016). In this study, we therefore compared the progression of DN in male and female UNx db/db C57BLKS mice using standard biochemical and histological endpoints, as well as renal cortex transcriptomic changes.

## 2 MATERIALS AND METHODS

### 2.1 Animals

All animal experiments were conducted in accordance with internationally accepted principles for the care and use of laboratory animals. The study was approved by the Danish Committee for Animal Research and covered by an institutional license issued to Tanja Xenia Pedersen (permit number: 2013‐15‐2934‐00843). Animals used in the studies were allowed to acclimatize for at least one week and kept in a light‐, temperature‐, and humidity‐controlled room (12‐hr light:12‐hr dark cycle; 22°C–24°C and 50% relative humidity). The animals had free access to standard chow diet and tap water.

Male and female C57BLKS/J db/db mice and male db/+ mice (JanVier) or BKS female db/+ mice (Charles River) arrived one week prior to surgery. At 7–8 weeks of age, db/db male and female mice were uninephrectomized (UNx), and sham‐operated db/+ male and female mice served as controls. *UNx surgery*. Mice were anesthetized by isoflurane inhalation, and a small incision was made to the left of the spine to expose the kidney. The left urethra, kidney artery, and kidney vein were identified and ligated with suture. Hereafter, the left kidney was removed. *Sham surgery*. For sham surgery, the kidney was exposed and gently manipulated, but not removed. For postoperative recovery, mice received 5 mg/kg enrofloxacin and 5 mg/kg carprofen administered subcutaneously for up to 2 days postsurgery. The mice were terminated 16 weeks after surgery, and the right kidneys were sampled for RNA sequencing, histology, and stereology.

### 2.2 Blood and urine analyses

Blood glucose was measured weekly for the first 4 weeks postsurgery and from there on every third week by collecting blood into heparinized glass capillary tubes and immediate suspension in glucose/lactate system solution buffer (EKF‐diagnostics). Blood glucose was measured using a BIOSEN c‐Line glucose meter (EKF‐diagnostics) according to the manufacturer's instructions. Glycated haemoglobin A1c (HbA1c) levels were measured at termination (16 weeks after surgery) using commercial kits (Roche Diagnostics) on a Cobas^TM^ C‐501 autoanalyzer (Roche Diagnostics). At study week 15, spot urine samples were collected directly from the penis/vulva to determine the urinary albumin‐to‐creatinine ratio (ACR). Urine creatinine was measured using the CREP2 kit (Roche Diagnostics) on a Cobas^TM^ C‐501 autoanalyzer. Urinary albumin was measured using a mouse albumin ELISA kit (Bethyl Laboratories, Inc.). Plasma blood urea nitrogen (BUN) levels were measured at termination (16 weeks after surgery) using commercial kits (Roche Diagnostics) on the Cobas^™^ C‐501 autoanalyzer.

### 2.3 Histology

At termination, the right kidney was dissected, weighed, divided sagitally, and one half was fixed in 10% neutral buffered formalin (4% formaldehyde, Hounisen) for 24 hr at room temperature. The fixed kidney tissue was cut transversely into 2 mm slabs using a systematic razor blade fractionator and a random starting position. The resulting 4–5 slabs were dehydrated and infiltrated with paraffin in a tissue processor (VIP5, Sakura) and then embedded with the cut surface down. A single 3 µm top section was cut on a microtome (Thermo Scientific) from each block representing a systematic uniform random sample of the half kidney.

#### 2.3.1 Immunochemical stain and stereological analyses

Periodic acid–Schiff (PAS) staining was performed using standard procedures. Briefly, kidney sections were deparaffinized and oxidized in 0.5% periodic acid (Sigma‐Aldrich). Next, sections were incubated with Schiff's reagent (Sigma‐Aldrich) and counterstained with Mayer's hematoxylin (Dako). Sections were dehydrated and mounted with Pertex (Histolab).

Stereological estimation of glomerular volume was determined using the Cavalieri principle of uniform random systematic sampling in combination with point counting (Gundersen, Jensen, Kiêu, & Nielsen, 1999). PAS‐stained slides were scanned under a 20× objective in an Aperio Scanscope AT slide scanner and imported into the stereology module of the Visiopharm Integrator Software (VIS, Visiopharm). An overlay grid system with an appropriate density was used to count all points hitting glomeruli.

#### 2.3.2 Immunohistochemical stains and quantification of fibrosis and glomerulosclerosis

Immunohistochemical (IHC) stains were performed using standard procedures. Collagen III IHC stain was used to quantify renal fibrosis. Kidney sections were deparaffinized. Antigen retrieval was performed by proteolytic digestion in 0.4% pepsin (Sigma‐Aldrich). Endogenous peroxidase activity was blocked in 1% H_2_O_2_ in TBS followed by protein blocking in blocking buffer (5% swine serum, 1% BSA, 0.05% Tween 20 in TBS). Subsequently, sections were incubated for one hour with goat anticollagen III antibody (Southern Biotech, cat. no. 1330‐01) diluted 1:100 in blocking buffer. The primary antibody was detected using Brightvision anti‐goat poly‐HRP system (Immunologic) and visualized with Liquid DAB + substrate chromogen system (Dako). Sections were counterstained in Gill's hematoxylin (Sigma‐Aldrich) prior to dehydration and mounting with Pertex.

Fluorescent IHC for double staining of podocin and collagen IV was performed to quantify the content of collagen IV inside glomeruli as a measure of glomerulosclerosis as follows: After deparaffinization, antigen retrieval was performed by boiling sections in TEG buffer (Tris‐EGTA pH 9, Sigma‐Aldrich) in a microwave oven for 30 min. Endogenous peroxidase activity was blocked in 1% H_2_O_2_ in TBS followed by protein blocking in blocking buffer. Subsequently, sections were incubated for 1 hour with primary antibody no. 1, rabbit anti‐podocin (cat. No. P0372, Sigma‐Aldrich) diluted 1:12,000 in blocking buffer for one hour. Sections were incubated with secondary antibody no. 1, HRP‐conjugated donkey anti‐rabbit (Jackson ImmunoResearch cat# 711‐036‐152) for 30 min followed by signal amplification using TSA plus FITC system (Perkin Elmer, NEL741B001KT). Subsequently, primary antibody no. 2, rabbit anticollagen IV (Novusbio cat# NB110‐59981) diluted 1:50 in blocking buffer, was added and sections incubated for one hour. Next, sections were incubated in secondary antibody no. 2, AlexaFluor 790‐conjugated donkey anti‐rabbit (Jackson ImmunoResearch cat# 711‐655‐152), for 30 min, counterstained with DAPI (Life technologies), and mounted with Prolong Diamond mounting medium (Life technologies).

Total kidney collagen III mass was determined using image analysis. Stained slides were scanned under a 20x objective in an Aperio Scanscope AT slide scanner and imported into an image analysis module in VIS. A Bayesian classifier was trained to detect DAB labeled collagen III and other tissue components. The total collagen III mass was calculated as the collagen III area fraction (area collagen III/total tissue area in sections) multiplied by the kidney weight.

Total glomerular collagen IV volume was determined similarly to collagen III mass. Slides were scanned under a 20× objective in an Olympus VS120 slide scanner with the appropriate fluorescent filters. The podocin‐positive FITC‐labeled area was used to identify all glomeruli. A simple threshold was used to detect AlexaFluor 790 labeled collagen IV inside glomeruli. Total collagen IV volume was calculated as the collagen IV area fraction (area collagen IV inside glomeruli/total glomerular area in sections) multiplied by the total glomerular volume as assessed by stereology.

### 2.4 RNA sequencing

RNA from snap‐frozen kidney samples (~15 mg per animal) stored at −80°C was extracted using the NucleoSpin^®^ 8 RNA kit (Macherey‐Nagel) and a vacuum manifold. The RNA quantity was measured using Qubit^®^ (Thermo Scientific), and RNA quality determined using a bioanalyzer with RNA 6,000 Nano kit (Agilent). About 500 ng purified RNA from each sample was used to generate a cDNA library using the NEBNext^®^ Ultra^™^ II Directional RNA Library Prep Kit for Illumina (New England Biolabs). cDNA libraries were sequenced (75 base‐pair, single end reads) on a NextSeq 500 using NextSeq 500/550 High Output Kit V2 (Illumina). Reads were aligned to the GRCm38 v89 Ensembl *Mus musculus* genome using STAR software (v.2.5.2a) (Dobin et al., 2013) with default parameters. A differential gene expression analysis was performed using the R package DEseq2 (Love, Huber, & Anders, 2014), and genes were considered significant at a false discovery rate (FDR) cut‐off of 0.01. A gene set analysis was conducted with the R package Piano (Väremo, Nielsen, & Nookaew, 2013) combined with gene annotation from the Reactome database (Haw & Stein, 2012).

### 2.5 Statistical analyses

Results are presented as mean ± standard error of mean (*SEM*). Two‐way analysis with Tukey's multiple comparisons test was used to test differences between groups. Urinary ACR values were log10‐transformed before group comparisons. Differences were considered statistically significant when *p* < .05.

## 3 RESULTS

### 3.1 Male and female UNx db/db mice display increased body weight, blood glucose, and HbA1c

**Figure 1:**
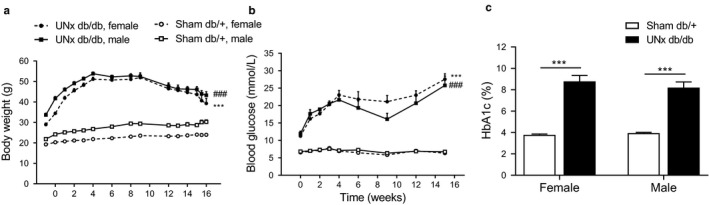
Male and female UNx db/db mice display increased body weight, blood glucose, and HbA1c indicating development of type 2 diabetes. (a) Body weight measured weekly in male and female sham db/+ and UNx db/db mice. Statistical analysis was performed on body weight measurements at week 16. (b) Blood glucose was assessed weekly from study start until week 4 and from there on every third week. Statistical analysis was performed on blood glucose measurements at week 15. ***p < .001 UNx db/db female versus Sham db/+ female; ^###^ *p* < .001 UNx db/db male versus Sham db/+ male. (c) Percentage of glycated hemoglobin A1c (HbA1c) was measured at termination 16 weeks after surgery. Values represent mean + *SEM* in Sham db/+, male (*n* = 9), UNx db/db, male (*n* = 12–16), Sham db/+, female (*n* = 8) and UNx db/db female (*n* = 10–14); ***p < .001 UNx db/db versus Sham db/+

Compared with sham‐operated db/+ controls, male and female UNx db/db mice displayed a substantial increase in body weight throughout the 16‐week study period, resulting in a significant body weight difference at termination (30.2 ± 0.9 Sham db/+ vs. 43.2 ± 1.7 g UNx db/db males, *p* < .001; 24.0 ± 0.4 Sham db/+ vs. 39.3 ± 2.3 g UNx db/db females, *p* < .001, Figure 1a). There was no statistically significant difference in body weight between UNx db/db males versus UNx db/db females (*p* = .36). Blood glucose was increased in male and female UNx db/db mice from the beginning of the study and remained significantly elevated until termination (Figure 1b). Correspondingly, HbA1c levels were significantly elevated in UNx db/db mice compared with sham db/+ controls 16 weeks postsurgery (4.0 ± 0.1 Sham db/+ vs. 8.2 ± 1.8% UNx db/db males, *p* < .001; 3.8 ± 0.1 Sham db/+ vs. 8.8 ± 0.6% UNx db/db females, *p* < .001, Figure 1c). For both blood glucose (*p* = .91) and HbA1c levels (*p* = .77), no significant change was found between UNx db/db males versus UNx db/db females.

### 3.2 Male and female UNx db/db mice display increased urine ACR, renal hypertrophy, and glomerulosclerosis

**Figure 2:**
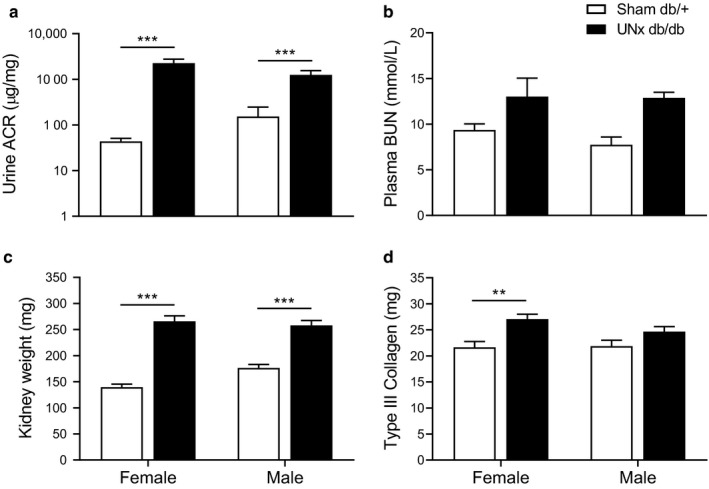
Male and female UNx db/db mice display renal hypertrophy and increased urine ACR. (a) Urine albumin‐to‐creatinine ratio (ACR) in spot urine samples collected 15 weeks after surgery. (b) Plasma BUN collected at termination (16 weeks after surgery). (c) Kidney weight (mg). (d) Total collagen III (mg) in the kidney was quantified using Visiopharm. Values represent mean + *SEM* in Sham db/+, male (*n* = 5–9), UNx db/db, male (*n* = 5–12), Sham db/+, female (*n* = 8) and UNx db/db female (*n* = 7–10); ***p < 00.1, **p < .01 UNx db/db versus Sham db/+

At study week 15, male and female UNx db/db mice displayed a significant increase in the urine ACR compared with controls (154 ± 92.8 Sham db/+ vs. 1,251 ± 306 µg/mg UNx db/db males week 15, *p* < .001; 44 ± 6.9 Sham db/+ vs. 2,263.8 ± 505 µg/mg UNx db/db females week 15, *p* < .001, Figure 2a), while no change was found between UNx db/db males versus UNx db/db females (*p* = .73). There was no significant increase in plasma BUN between UNx db/db mice and db/+ controls of the same sex (7.7 ± 0.9 Sham db/+ vs. 12.9 ± 0.6 mmol/L UNx db/db males, *p* = .11; 9.4 ± 0.7 Sham db/+ vs. 13.0 ± 2.0 mmol/L UNx db/db females, *p* = .29, Figure 2b), neither between males and females UNx db/db mice 16 weeks postsurgery. At termination, male and female UNx db/db mice displayed renal hypertrophy as indicated by significantly increased kidney weight compared with sham db/+ controls (176 ± 6.7 Sham db/+ vs. 258 ± 9.5 mg UNx db/db males, *p* < .001; 140 ± 5.5 Sham db/+ vs. 266 ± 10.5 mg UNx db/db females, *p* < .001, Figure 2c). Four sham‐operated male db/+ were excluded at the time of termination due to hydronephrosis, whereas none of the female db/+ or UNx db/db mice developed hydronephrosis. Renal collagen III levels were significantly increased in female UNx db/db mice compared with female sham db/+ controls (21.7 ± 1.1 Sham db/+ vs. 27.1 ± 0.9 mg UNx db/db females, *p* < .01, Figure 2d), whereas no statistically significant change was found in male UNx db/db mice versus male sham db/+ controls (*p* = .35). For UNx db/db males versus UNx db/db females, no difference was found in kidney weight (*p* = .91) and renal collagen III levels (*p* = .29).

**Figure 3:**
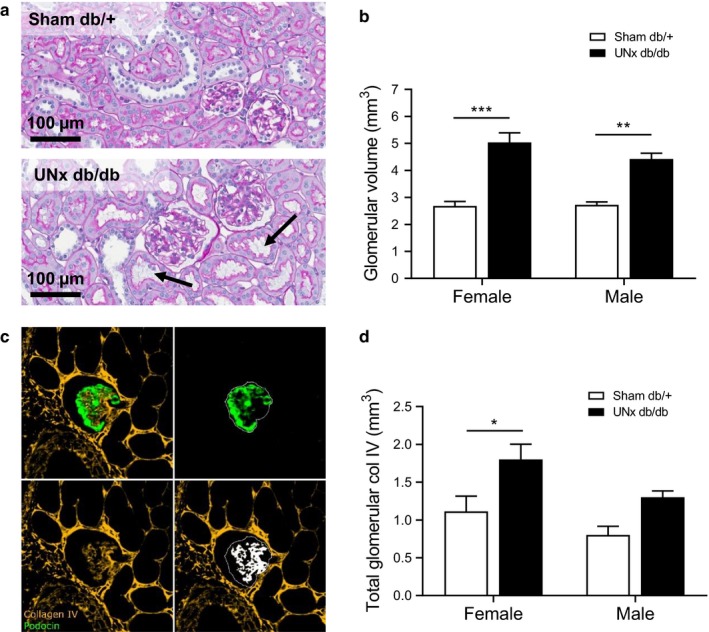
Increased glomerular volume and glomerulosclerosis in UNx db/db mice. (a) Representative images of PAS‐stained kidney sections from female Sham db/+ and UNx db/db mice. The arrows represent dilated tubules. Scale bar 100 µm. (b) Total glomerular volume quantified by stereology on PAS‐stained kidney sections at week 16. (c) A double immunohistochemical stain for podocin (green) and collagen IV (yellow) was performed to quantify glomerulosclerosis (white overlay). (d) Total glomerular collagen IV was quantified using stereology on podocin/collagen IV double stained kidney sections at week 16. Values represent mean + *SEM* in Sham db/+, male (*n* = 5), UNx db/db, male (*n* = 12), Sham db/+, female (*n* = 5–8) and UNx db/db female (*n* = 10); *p < .05, **p < .01, ***p < .001 UNx db/db versus Sham db/+

Representative images of PAS‐stained renal cortex from female sham db/+ and UNx db/db mice 16 weeks after surgery demonstrated increased incidence of tubular dilation and glomerular hypertrophy in UNx db/db kidneys (Figure 3a). Stereological quantitation revealed increased total glomerular volume in both male and female UNx db/db mice compared with their respective controls (2.7 ± 0.1 Sham db/+ vs. 4.4 ± 0.2 mm^3^ UNx db/db males, *p* < .01; 2.7 ± 0.2 db/+ Sham vs. 5.0 ± 0.4 mm^3^ UNx db/db females, *p* < .001, Figure 3b). In addition, no difference was found in total glomerular volume in UNx db/db males versus UNx db/db females (*p* = .27). Assessment of glomerulosclerosis (Figure 3c) was performed by double immunohistochemistry for podocin (podocytes) and collagen IV (fibrosis). The podocin stain was used to identify glomeruli with intraglomerular collagen IV as a proxy for glomerulosclerosis. Quantitative image analysis indicated a significant increase in glomerular IV collagen in UNx db/db females compared with sham db/+ females (1.1 ± 0.2 Sham db/+ vs. 1.8 ± 0.2 mm^3^ UNx db/db females, *p* < .05). In male mice, glomerular collagen IV was, however, not increased in UNx db/db compared with sham db/+ controls (0.8 ± 0.1 Sham db/+ vs. 1.3 ± 0.1 mm^3^ UNx db/db males, *p* = .088, Figure 3d). There was no significant change in glomerular collagen IV in UNx db/db males versus UNx db/db females (*p* = .07).

### 3.3 The kidney transcriptome of male UNx db/db mice versus Sham db/+

**Figure 4:**
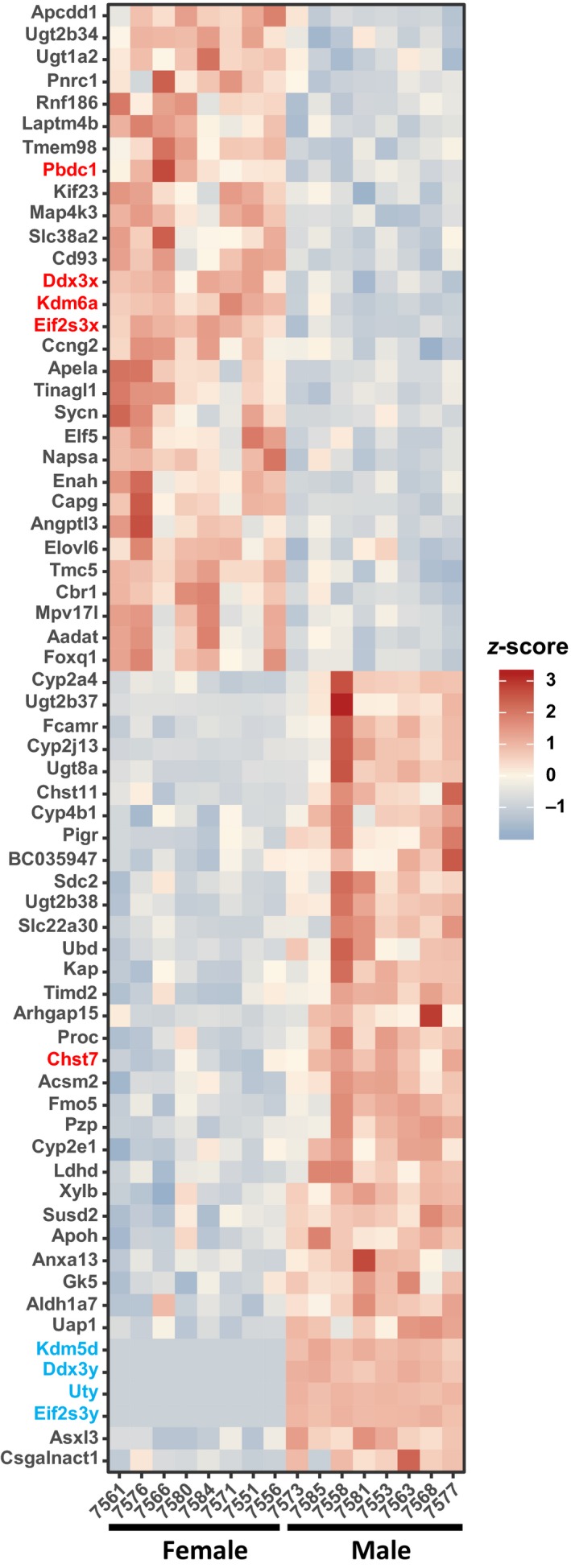
Transcriptional changes in the cortical kidney between male and female UNx db/db mice. The heat map shows the log2 fold change of significantly regulated genes (*p* < .01) between male and female UNx db/db mice. Gene names highlighted in red color are located on the X chromosome, while gene names highlighted in blue color are located on the Y chromosome. UNx db/db, male (*n* = 8) and UNx db/db female (*n* = 8)

To further explore sex‐associated differences in the kidney, we compared gene expression patterns in renal cortex between male and female UNx db/db mice using RNA sequencing. Surprisingly, we uncovered only 66 genes that were differentially expressed between male and female UNx db/db mice out of the whole transcriptome (~22.000 genes) (Figure 4). Of these genes, 30 genes were upregulated in female UNx db/db mice and 36 genes were upregulated in male UNx db/db mice. None of the 66 identified genes have known associations with DN susceptibility or progression. Interestingly, 9 of the genes regulated (~14% of the total differentially expressed genes) are located on the sex chromosomes. On the X chromosome, *Pbdc2*, *Ddx3x*, *Kdm6a*, and *Eif2s3x* were upregulated in female UNx mice, whereas *Chst7* was upregulated in male UNx db/db mice (Figure 4). The regulated *Kdm5d*, *Ddx3y*, *Uty,* and *Eif2s3y* were all restricted to the Y chromosome (Figure 4). A gene set enrichment analysis was used to identify overrepresented gene sets in either male or female UNx db/db mice. No significantly enriched pathways were identified, suggesting that the progression of DN in males and females is also highly similar on the mRNA level.

## 4 DISCUSSION

The db/db mouse is one of the most widely used animal models of diabetes and presents with renal complications (Tesch & Lim, 2010). Here, we provide a direct comparison of the DN severity/phenotype in male and female UNx db/db mice 16 weeks after surgery, to assess potential sex specific differences of high value for preclinical and clinical studies. Based on a series of state of the art biochemical, histological, and transcriptional analyses, we demonstrate that the DN phenotype is highly conserved across sex.

Recently, the National Institutes of Health has required that grant applications account for sex as a biological variable in preclinical research (Clayton & Collins, 2014) emphasizing the importance and urgency of addressing sex differences in research. For years, a serious male bias may have obscured potential key sex differences in clinical trials (Beery, 2018), as illustrated by the poorer treatment outcomes for women with female patients experiencing higher rates of adverse drug events than men (Franconi, Brunelleschi, Steardo, & Cuomo, 2007). Traditionally, the bias toward male subjects have centered around a concern for confounding contribution from the estrous cycle. However, empirical studies and analyses addressing these concerns in several rodent species have now demonstrated that females may not be more variable than males (Beery, 2018; Prendergast, Onishi, & Zucker, 2014).

The C57BLKS db/db mouse is one of the most widely used animal models of diabetes with renal complications (Tesch & Lim, 2010), as substantiated by albuminuria and mesangial matrix expansion (Harlan et al., 2018; Heuer et al., 2017; Tang et al., 2017). In agreement with the literature, the present study demonstrated albuminuria and glomerular hypertrophy in the UNx db/db model. Both male and female UNx db/db mice showed similar progressive changes in body weight, hyperglycemia, and HbA1c, indicating a comparable diabetic phenotype between sexes. UNx did not influence the severity and progression of the diabetic phenotype in UNx db/db mice when compared with nonoperated db/db mice (Cohen et al., 2001; Lee & Bressler, 1981; Terami et al., 2014). Furthermore, similar levels of renal and glomerular hypertrophy were observed in male and female UNx db/db mice, along with increased urine ACR. Male and female UNx db/db mice presented with similar plasma BUN levels, and no significant differences were found between UNx db/db mice and controls within the same sex either indicating only mild kidney disease in the UNx db/db mouse. Although renal collagen III was only significantly increased in female UNx db/db mice only, male UNx db/db mice also showed elevated levels of renal collagen III. In comparison, costaining with podocin and collagen IV was used to assess glomerulosclerosis, as glomerular collagen IV has been associated with the development of glomerulosclerosis in preclinical and clinical studies (Ikeda et al., 1991; Lee et al., 1998; Tamsma et al., 1994). Glomerular collagen IV differed modestly between male and female UNx db/db mice, with female mice exhibiting significantly increased intraglomerular collagen IV. However, male mice also exhibited a strong tendency toward glomerulosclerosis. Therefore, while subtle sex differences exist in the UNx db/db model, no physiologic meaningful differences were observed in the DN phenotype of male versus female mice in this present study.

To explore the influence of sex on the renal transcriptome, we performed kidney cortex RNA sequencing and revealed that only 66 genes were significantly regulated between male and female UNx db/db mice. None of the 66 identified genes have known associations with DN susceptibility or progression. The X chromosome located gene, *Chst7*, found to be upregulated in male UNx db/db mice, encodes the enzyme carbohydrate sulfotransferase 7. The enzyme catalyzes the transfer of sulfate groups to N‐acetylgalactosamine, and sulphated N‐acetylgalactosamine have been implicated in many biological processes including extracellular matrix deposition (Kitagawa, Fujita, Ito, & Sugahara, 2000), suggesting that this gene might be implicated in DN progression. The genes *Eif2s3x* and *Ddx3x*, located on the X chromosome and upregulated in female UNx db/db mice, are homologues to the Y‐linked genes *Eif2s3y* and *Ddx3y* upregulated in male UNx db/db mice, underlining the similar DN phenotype of the two sexes.

Overall, our observations are in agreement with a similar study Ma et al. (2019) comparing male and female eNOS^−/−^ db/db mice and concluding that no sex difference exists in renal structural and functional injury. In a different study Østergaard et al. (2016), investigating the influence of mouse strain on the susceptibility to kidney injury, differences in body weight, blood glucose, and plasma insulin levels were shown between male and female DBA/2J db/db mice. However, these differences in the diabetic phenotype did not translate into differences on the development of urinary ACR levels. Overall, studies indicate that sex does not exert a major impact on the susceptibility to or progression of DN in the db/db mouse model. In accordance to previous reports (Ninomiya, Inomata, & Ogihara, 1999; Springer et al., 2014), we observed several cases of hydronephrosis in male mice exclusively. The susceptibility to hydronephrosis and pyelonephritis (Harlan et al., 2018; Springer et al., 2014) in male db/db mice may pose an argument for selecting female over male mice to further refine the db/db model of DN. However, all together, data do not support a biological rationale for using male or female mice only in preclinical research and drug development in DN. Like most animal models of DN, the UNx db/db mouse does not capture the functional glomerular decline associated with severe progression of DN (Heuer et al., 2017; Zhou, Cheung, Liu, & Huang, 2014). The lack of mouse models that exhibit the spectrum of pathological renal changes during DN progression has been a significant impediment for the development of new treatments. This may mainly be driven by the absence of hypertension during late stage DN, which is the main risk factor associated with progression of DN to ESRD (Betz & Conway, 2014; Conway et al., 2011; Heerspink & Zeeuw, 2011). The use of an adeno associated virus vector to overexpress the renin gene in the db/db UNx mouse model offers an intriguing means of adding this hypertensive component to both accelerate and progress the disease phenotype to an advanced stage of DN (Harlan et al., 2018).

In conclusion, the UNx db/db model recapitulates hallmarks of early‐stage DN and can be used to study aspects of initial disease progression. In addition, our results suggest that both male and female UNx db/db mice can be used in future DN studies, as only subtle sex differences were found.

## CONFLICT OF INTEREST

All authors are current or previous employees of Gubra ApS.

## AUTHOR CONTRIBUTIONS

L.N.F., T.X.P., T.S., J.J., and N.V. designed the study; F.S.E. and T.J. performed experiments; F.S.E., L.N.F., T.J., T.S., S.T.T., and J.C.N. analyzed data; F.S.E., L.N.F.,T.J., T.S., T.X.P., J.J., and M.V.Ø. interpreted results of experiments; F.S.E. and J.C.N. prepared figures; F.S.E., B.B.B., and T.X.P. drafted manuscript; F.S.E., B.B.B., T.X.P., J.J., and M.V.Ø. edited and revised manuscript; L.N.F., K.F., N.V., and J.J. approved final version of manuscript.
